# Emphysematous Cystitis in an Uncontrolled Diabetic: A Case Report

**DOI:** 10.7759/cureus.20328

**Published:** 2021-12-10

**Authors:** Shelby A Swede, Adedolapo O Ojo, Tobe Momah

**Affiliations:** 1 Family Medicine, University of Mississippi Medical Center, Jackson, USA; 2 Surgery, Icahn School of Medicine at Mount Sinai/Elmhurst Hospital Center, New York, USA

**Keywords:** diabetes, urinary tract infection, air in bladder wall and lumen, pneumaturia, emphysematous cystitis

## Abstract

Emphysematous cystitis is a rare potentially life-threatening infection of the urinary tract system commonly caused by gas forming micro-organisms like *Escherichia coli*. It is commonly seen in diabetics and middle-aged-elderly women. Presenting symptoms could be variable and unusual on many occasions. Therefore, a high index of suspicion is crucial in prompt/accurate diagnosis and treatment of this disease. This case report presents emphysematous cystitis in a middle-aged woman with poorly controlled diabetes mellitus. Pneumaturia and evidence of emphysematous cystitis on computerized tomography were also noted in this case.

## Introduction

Emphysematous cystitis (EC) is a rare and severe form of urinary tract infection (UTI) characterized by gas within the lumen and wall of the bladder [1,2]. Numerous micro-organisms have been implicated in the etiology of EC, but the most common ones have been *Escherichia coli* and *Klebsiella pneumoniae* [2,3]. EC is most often seen in diabetic middle-aged/elderly women [2]. It is a potentially life-threatening condition with a variable patterns of clinical presentation; therefore, there is a need for prompt evaluation and management to achieve better health outcomes [1,3].

## Case presentation

A 64-year-old African-American female with a history of poorly controlled type 2 diabetes with retinopathy, hypertension, hyperlipidemia, myocardial infarction, coronary artery disease with stent replacement, chronic kidney disease stage 3b, gastritis and esophagitis presented to the ED with nausea, vomiting and severe heartburn for five days unrelieved with antacid medication. The patient described the pain as burning and radiating to the subscapular region with intermittent, non-radiating left lower quadrant pain, chills and passage of urine that appeared cloudy and frothy with a distinct color. Of note, the patient-reported her last HbA1C to be 11.7%.

**Figure 1:**
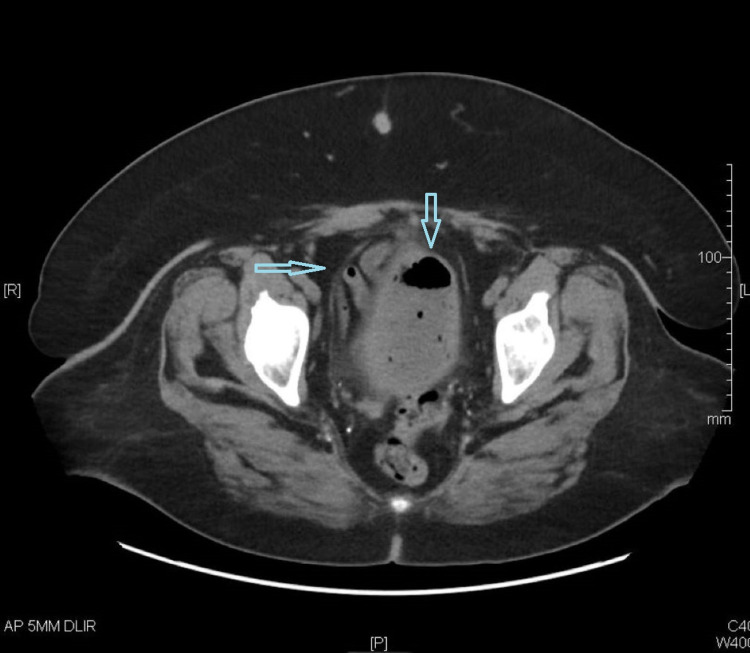
Abdomen-pelvis computerized tomography showing gas in the bladder wall and lumen. The two arrows are pointing at gas under the bladder wall.

On physical examination, the patient was noted to appear mildly uncomfortable with a soft, non-distended abdomen and moderate suprapubic tenderness. She was afebrile, hemodynamically stable, and laboratory testing results were as follows: white blood cell count 9,800/uL, HbA1C 10%, blood glucose 251 mg/dL, blood urea nitrogen 23 mg/dL, and creatinine 2.1 mg/dL. Urinalysis showed small leukocyte esterase, 45 white blood cells per high power field, and >10,000 bacteria. Computerized tomography of the abdomen-pelvis revealed gas in the bladder wall and lumen, and thickening of the bladder wall (Figure 1).

Patient was given 1 g IV Rocephin, 0.9% normal saline at 150 cc/hr, admitted to the floor, and had clinical and symptomatic improvement within 24 hours of medical management.

After admission, urine cultures grew *E. coli* and the patient was started on IV Zosyn 2.25 g every six hours after obtaining sensitivities, which was transitioned to per oral (PO) Augmentin 500-125 mg twice daily for discharge and advised to follow-up outpatient for further management.

## Discussion

Emphysematous cystitis (EC) is an infrequently seen severe infection distinguished by air within the urinary bladder wall and lumen [1,2]. It is mostly seen in middle-aged or elderly diabetic women [2,3]; other common risk factors are a history of chronic urinary tract infections (UTIs), presence of indwelling catheters, obstructive uropathy, neurogenic bladder, and immunosuppression [1,2].

Diabetes is noted to be the strongest risk factor for EC, with 70% of EC patients being diabetic. EC occurs in diabetics because of a combination of factors peculiar to them; high glucose levels (serves as a substrate for microbes to produce carbon dioxide through fermentation), diabetic nephropathy, bladder dysfunction from diabetic neuropathy, renal artery stenosis, and impaired leucocyte function [3,4]. The most commonly isolated pathogens from the urine of EC patients are *E. coli*, *K. pneumoniae*, and Enterobacter [1,3].

The clinical presentation of EC has been found to be variable, ranging from being asymptomatic to severe sepsis [1-3]. Most patients with symptoms have abdominal pain, hematuria, ischuria, and pneumaturia [1,3]. Other possible symptoms are fever, dysuria, urgency, and frequency [3]. As a condition that is potentially life-threatening and with highly varied and non-specific symptoms, a high index of suspicion is required with prompt management [2].

Irrespective of presenting symptoms, the use of radiologic imaging is crucial for the definitive diagnosis of EC. Plain abdominal x-rays (most commonly used) usually show rim(s) of gas lucency in the bladder wall and/or lumen. A CT scan is more sensitive and shows severity, extent and differentiates EC from other conditions with air in the abdomen/pelvis like vesicocolic fistula, vesicovaginal fistula, uterine gas gangrene, trauma, intra-abdominal abscesses, etc [3,4].

Urinalysis, urine culture and gram staining are also essential for isolating the offending organism and guiding antibiotic therapy. Tight glycemic control and early bladder catheterization (enables bladder rest and monitoring of urine output) are also crucial in EC management [3].

Our patient is a middle-aged woman with poorly controlled diabetes who had left lower quadrant pain and pneumaturia. Her poorly controlled diabetes may have predisposed her to EC. Prompt diagnosis, investigation with urinalysis, urine culture, CT of abdomen and pelvis and treatment with IV fluids, tight glycemic control, and antibiotics were critical to curtailing the progression of the infection. Our patient responded well to intravenous antibiotics and is currently stable. Prompt management was crucial to preventing the progression of the condition to more severe conditions like bladder necrosis, emphysematous pyelonephritis, and urosepsis [4].

## Conclusions

Emphysematous cystitis is an infrequently seen condition, which can be asymptomatic, have specific clinical manifestations like pneumaturia or a variety of other non-specific manifestations. Diabetes is a common predisposing factor and prompt diagnosis and management are vital to achieving better health outcomes and curtailing the progression of the condition to more severe conditions like bladder necrosis, emphysematous pyelonephritis, and urosepsis.
